# Electrical characterization of multi-gated WSe_2_/MoS_2_ van der Waals heterojunctions

**DOI:** 10.1038/s41598-024-56455-x

**Published:** 2024-03-09

**Authors:** Phanish Chava, Vaishnavi Kateel, Kenji Watanabe, Takashi Taniguchi, Manfred Helm, Thomas Mikolajick, Artur Erbe

**Affiliations:** 1 Institute of Ion Beam Physics and Materials Research, Helmholtz Zentrum Dresden-Rossendorf, 01328 Dresden, Germany; 2 Faculty of Electrical and Computer Engineering, Technische Universität Dresden, 01062 Dresden, Germany; 3 Research Center for Electronic and Optical Materials, National Institute for Materials Science, 1-1 Namiki, Tsukuba, 305-0044 Japan; 4 Research Center for Materials Nanoarchitectonics, National Institute for Materials Science, 1-1 Namiki, Tsukuba, 305-0044 Japan; 5 NaMLab gGmbH, 01187 Dresden, Germany

**Keywords:** Electronic devices, Two-dimensional materials, Electrical and electronic engineering, Electronic properties and materials

## Abstract

Vertical stacking of different two-dimensional (2D) materials into van der Waals heterostructures exploits the properties of individual materials as well as their interlayer coupling, thereby exhibiting unique electrical and optical properties. Here, we study and investigate a system consisting entirely of different 2D materials for the implementation of electronic devices that are based on quantum mechanical band-to-band tunneling transport such as tunnel diodes and tunnel field-effect transistors. We fabricated and characterized van der Waals heterojunctions based on semiconducting layers of WSe_2_ and MoS_2_ by employing different gate configurations to analyze the transport properties of the junction. We found that the device dielectric environment is crucial for achieving tunneling transport across the heterojunction by replacing thick oxide dielectrics with thin layers of hexagonal-boronnitride. With the help of additional top gates implemented in different regions of our heterojunction device, it was seen that the tunneling properties as well as the Schottky barriers at the contact interfaces could be tuned efficiently by using layers of graphene as an intermediate contact material.

## Introduction

Two-dimensional (2D) materials can be easily isolated from their bulk counterparts owing to relatively weak out-of-plane van der Waals (vdW) forces between each layer. This property not only allows for obtaining highly crystalline ultra-thin layers simply by mechanical exfoliation^1^ but also provides an excellent basis for stacking different materials in order to form heterostructures with tailored properties^2^. The family of 2D materials offers materials with a wide range of unique electronic and opto-electronic properties and, therefore, integrating different types of 2D materials into heterojunctions opens the door to the realization of several multi-functional (opto-) electronic devices^3–5^. The major advantage of 2D material-based heterojunctions arises from the possibility of the formation of atomically sharp junctions, with very low density of interfacial defects as compared to epitaxially grown bulk heterojunctions. Achieving an abrupt junction with minimal defects is crucial for electronic devices that are based on band-to-band tunneling mechanism like tunnel diodes and tunnel field-effect transistors^6^. Although recent experimental studies on 2D heterojunction devices constructed using various material systems^7–22^ provide a quantitative analysis in terms of diode or transistor performance, a deeper qualitative understanding of the transport mechanisms in the heterojunction is often missing. In this study, we investigate a 2D semiconductor heterojunction by characterizing the electrical transport down to cryogenic temperatures using different device architectures and gating schemes. As WSe_2_ and MoS_2_ are the most stable^23^ and widely studied 2D semiconductor materials, we chose to fabricate our devices based on a WSe_2_/MoS_2_ junction.

Previous reports of WSe_2_/MoS_2_ based tunneling devices involved using a combination of high-k dielectric^7,10,24,25^ or ion gel dielectric^9^ to achieve an efficient electrostatic control or even chemical functionalization^8,10^ to tune the doping levels of individual materials in the heterojunction to improve the tunneling currents. Most of these studies also employed different contact metals on either side of the heterojunction to achieve a better carrier injection, which increases the complexity of device fabrication. In contrast, all our devices are fabricated by using a common contact and encapsulation scheme^10,26–28^ using layers of graphene and hexagonal-boronnitride (h-BN), which not only eases the device fabrication process by minimizing the lithography steps but also reduces the substrate-induced scattering effects on the active channel. Moreover, our process can be adopted for investigating transport in heterojunctions based on other material systems which could be air-sensitive.

In addition to our previous study on dual-gated WSe_2_/MoS_2_^28^, we propose here a triple gate architecture in order to evaluate the local electrical behavior in different parts of the heterojunction. We observed band-to-band tunneling current in our devices which is supported by the presence of negative differential resistance (NDR) at low temperatures. We then characterized the devices extensively under various bias conditions and found that the Schottky barrier at the contact interface plays a crucial role in improving the magnitude of the tunneling current in our device, while the steepness of switching is still limited by the low-$\kappa$ hBN dielectric environment. The additional gates implemented make the device quite versatile in terms of specific electronic applications owing to the tunability offered by programming the gates selectively.

## Fabricated devices

**Figure 1:**
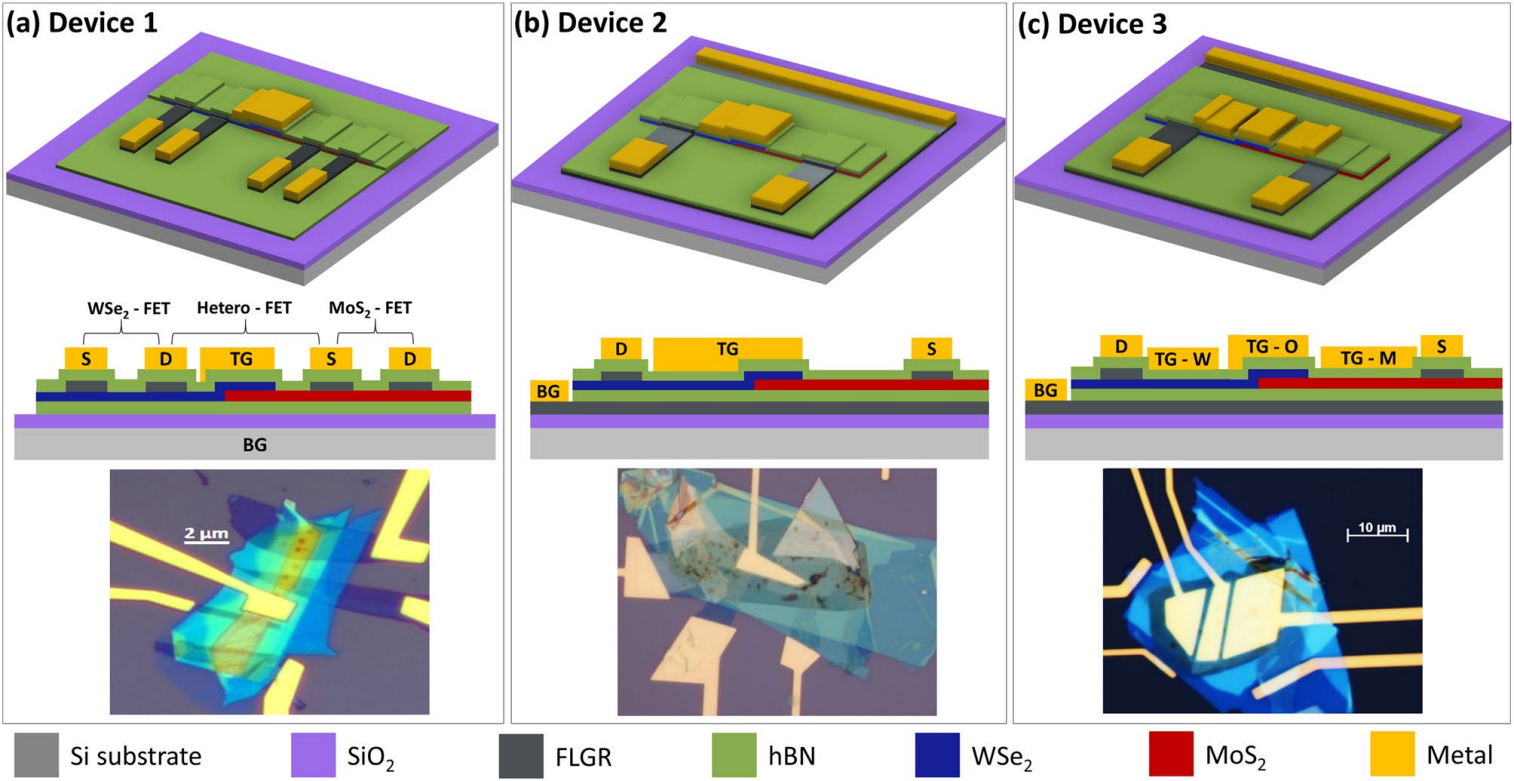
Schematics of fabricated devices along with corresponding optical micrographs (**a**) ‘Device 1’: Single top gate device with thick equivalent oxide thickness (EOT); (**b**) ‘Device 2’: Single top gate device with thin EOT; (**c**) ‘Device 3’: Multi-gated device with thin EOT. The color legend at the bottom indicates different materials in the schematics.

We used mechanically exfoliated^1^ flakes of different 2D materials to fabricate our heterojunction devices in different configurations and classify them mainly into three device types based on the implementation of gates as shown in Fig. 1. For all the device types, the semiconducting heterojunction is composed of WSe_2_ and MoS_2_ with thicknesses ranging from 3 nm to 10 nm. The exact thicknesses of the 2D flakes used for each device are mentioned in Table 1 of the supplementary information. For transition metal dichalcogenides (TMD) having such a thickness range, the band structure is much closer to that of their bulk counterparts rather than their mono-layer form^29^. Semi-metallic few-layered graphene (FLG) is used throughout all the devices as an intermediate contact material between the TMD and the metal electrodes. The use of graphene allows to minimize the effect of Fermi level pinning of the metal to the semiconductor bands^30^. Additionally, the entire heterojunction region is encapsulated from top and bottom with hexagonal-boronnitride (h-BN) in such a way that small portions of graphene flakes are not covered by the top h-BN in order to establish an electrical connection to the metal electrodes. The thicknesses of the h-BN flakes used range from 5 to 15 nm. The idea behind using h-BN encapsulation is twofold. Firstly, h-BN offers sharp van der Waals interfaces with the semiconducting channel which helps in minimizing the impurity scattering and coulomb interactions^31^. Secondly, this type of encapsulation scheme lays the foundation for investigating similar devices with relatively unstable materials that are prone to surface oxidation and degradation in air. We, therefore, use this general device structure commonly among all our device configurations, where the active channel materials, electrical contacts, and the dielectric are stacked entirely by using different two-dimensional materials.

For ‘Device 1’ (Fig. 1a), the above-mentioned general stack is fabricated on a highly p-doped silicon substrate with a thermally grown oxide ($\text {SiO}_{2}$) having a thickness of 285 nm. The doped silicon substrate serves as the global bottom gate for the entire device, while a top gate is patterned using electron beam lithography in such a way that it covers only the WSe_2_ side of the junction along with the region consisting of the WSe_2_/MoS_2_ overlap. For the global bottom gate, the thickness of the bottom h-BN should be accounted for in the dielectric thickness. The total dielectric stack amounts to an equivalent oxide thickness (EOT) of about 300 nm. Additional graphene layers are used on both WSe_2_ and MoS_2_ flakes in order to characterize individual flakes by excluding the junction region as indicated by the schematic in Fig. 1a, where three distinct transistors can be measured using the same heterojunction device.

In the case of ‘Device 2’, an additional graphene layer spanning laterally throughout the device is used between the bottom h-BN and the substrate (Fig. 1b). This graphene layer acts as the global bottom gate electrode and therefore eliminates the requirement of a substrate with a thick oxide as in the case of ‘Device 1’. Therefore, the EOT of the bottom gate dielectric in this case solely corresponds to the thickness of the bottom h-BN layer and is independent of the substrate or an oxide layer grown on it. Moreover, the silicon substrate used for this device could be potentially replaced by a flexible substrate for specific applications. The top gate here is designed similarly to that of ‘Device 1’. The use of this graphene layer as a gate electrode makes it extremely challenging to use additional graphene layers on MoS_2_ and WSe_2_ flakes as done for ‘Device 1’. Therefore, the individual characteristics of MoS_2_ and WSe_2_ are not investigated for this device configuration.

Lastly, for ‘Device 3’, a similar stack is fabricated as in the case of ‘Device 2’, but instead of just having one top gate on the WSe_2_ side of the channel, three different top gates are designed so that each of the top gates modulates different regions of the entire channel (Fig. 1c), i.e part of the WSe_2_ flake extending out to the left without overlap to MoS_2_, the actual overlap region of the WSe_2_ and MoS_2_ flakes, and finally the MoS_2_ flake extending to the right of the overlap region. For the electrical measurements, only one of the three top gates is active while the other two are connected to ground.

## Results and discussion

**Figure 2:**
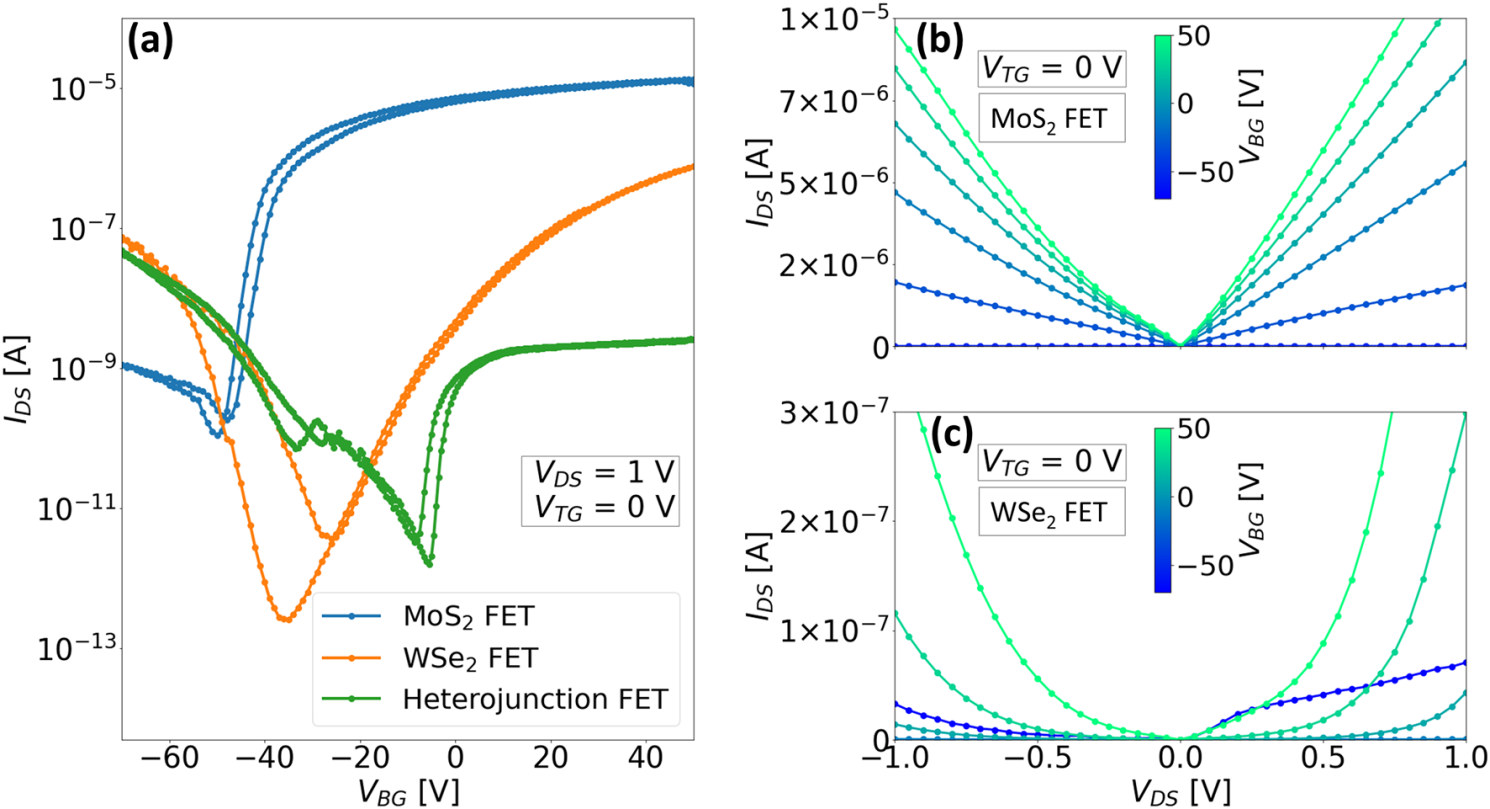
Room temperature electrical characteristics of ‘Device 1’: (**a**) Individual transfer characteristics of WSe_2_, MoS_2_ and WSe_2_/MoS_2_ transistors measured by sweeping the bottom gate; Output characteristics of (**b**) MoS_2_ and (**c**) WSe_2_ transistors respectively.

Figure 2a shows the measured room temperature transfer characteristics of the three distinct transistors embedded within ‘Device 1’, i.e MoS_2_-FET, WSe_2_-FET and the heterojunction FET (HJ-FET). The transfer curves were obtained by sweeping the global bottom gate at a constant drain-source voltage ($V_{DS}$) of 1 V and a 0 V bias on the top gate. A clear modulation by the bottom gate is observed for all three transistors, with ambipolar curves for the WSe_2_-FET and HJ-FET, while n-type characteristic is observed for the MoS_2_-FET with a much higher off-current (defined as the minimum current observed in the transfer curve here, since the threshold voltage of the device is not yet optimized for a practical application) which indicates a high n-type doping density. All three transistors exhibit a significant negative threshold voltage owing to the traps and fixed charges in the thick $\text {SiO}_{2}$ dielectric. Similar characteristics have also been reported previously^25,27,32^ for MoS_2_, WSe_2_ and the heterojunction transistors that were measured using a doped substrate coupled with an oxide dielectric layer as the gate stack. However, the ambipolar nature of the heterojunction transfer curve as well as the appearance of a negative transconductance (NTC) peak varies widely over different studies based on WSe_2_/MoS_2_ junctions^25,27,33,34^. In our device, we obtain an ambipolar curve with a small peak centered around $V_{BG}=-30$ V (green curve in Fig. 2a). The position of this peak is around the same voltage as the inversion point of the WSe_2_-FET, which shows that the carrier concentration in the WSe_2_ flake influences the peak position. This has also been demonstrated by Yang et al.^27^ in WSe_2_/MoS_2_ junctions, where both the occurrence and the position of the NTC peak can be tuned with the help of additional gates to modulate the carrier densities in the junction. Moreover, it also greatly depends on the relative vertical orientation of the stack, for example, when MoS_2_ is on top of WSe_2_, the effect of the bottom gate on the MoS_2_ layer is screened if the WSe_2_ layer is thicker. In this case, the bottom gate has a much more effective modulation of the WSe_2_ flake and the NTC peak is therefore more prominent with peak-to-valley ratios of several orders of magnitude^33,34^.

The output characteristics of the MoS_2_ and the WSe_2_ transistors are shown in Fig. 2b and c respectively at different steps of bottom gate voltage while the top gate was grounded ($$V_{TG} = 0 $$ V). It can be seen that for the MoS_2_ transistor, the output curves are nearly linear indicating a good graphene–MoS_2_ contact. However, for the case of WSe_2_ transistor, the characteristics are highly non-linear at much lower current levels which suggests the presence of a much higher barrier at the graphene–WSe_2_ interface. Usually, metals with high work function like platinum or palladium have been shown to reduce the Schottky barrier height (SBH) for holes in WSe_2_^35^, which results in a unipolar p-type characteristic. This has also been implemented for heterojunctions by using dissimilar metals for contacting MoS_2_ and WSe_2_ in order to obtain nearly Ohmic contacts to both flakes^24,25,36^. However, we used graphene for both MoS_2_ and WSe_2_ in order to employ the encapsulation scheme using h-BN and avoid any etching steps. This not only eases the fabrication process but also reduces the possibility of contamination or defects that arise from any additional processing steps. We also extracted the SBH at the graphene/MoS_2_ and graphene/WSe_2_ interfaces and found the values to be about 60 me V for MoS_2_ and 180 me V for WSe_2_ (Supplementary information-S1).

**Figure 3:**
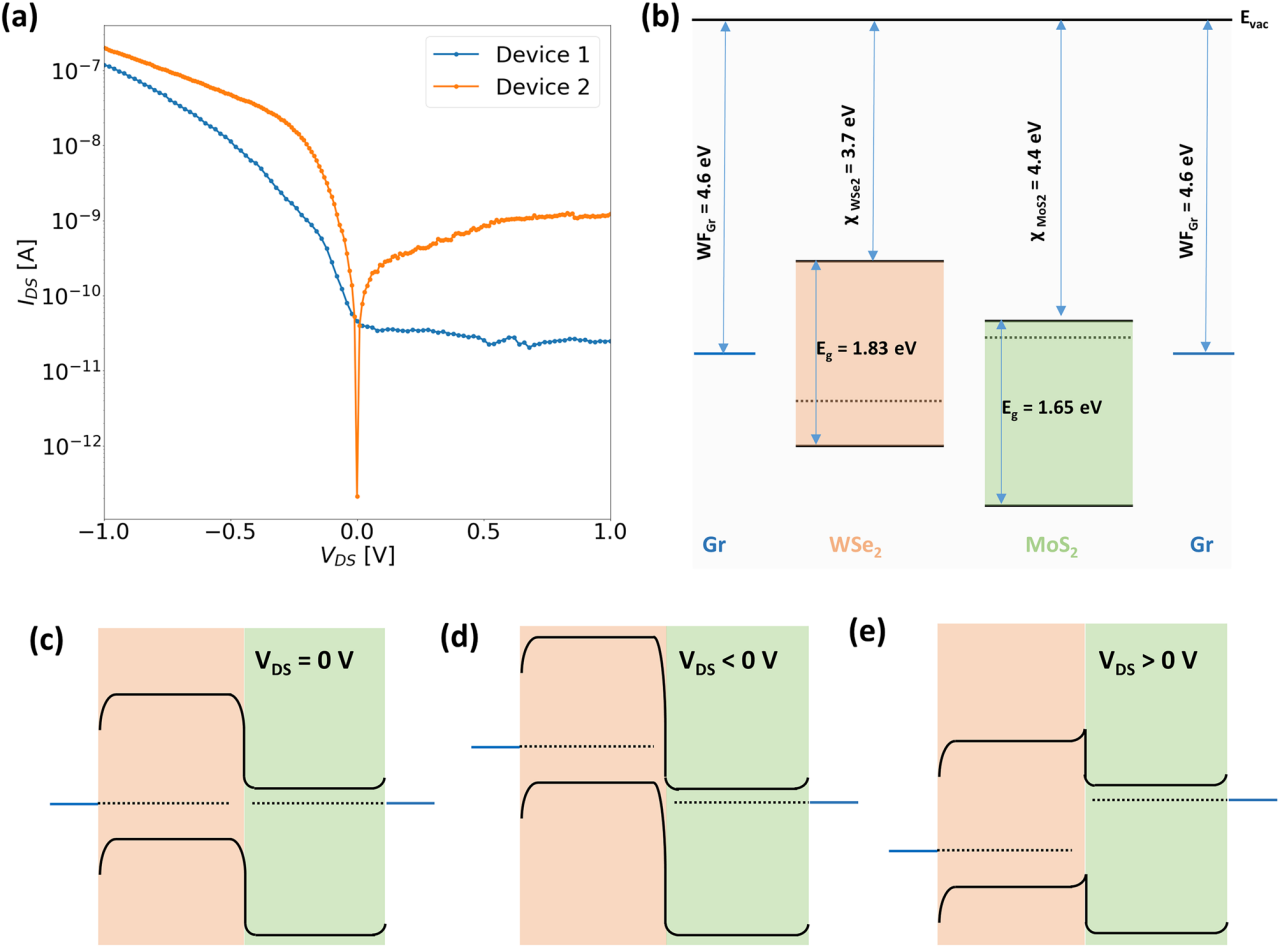
(**a**) Room temperature output characteristics (semi-logarithmic) of ‘Device 1’ and ‘Device 2’ plotted together with both the gates grounded ($$V_{TG} = V_{BG} = 0 $$ V) (**b**) Alignment of graphene Work function to the energy bands of WSe_2_ and MoS_2_ with respect to the vacuum level; Simplified energy band diagrams of the heterojunction (**c**) at thermal equilibrium (**d**) under reverse bias (**e**) under forward bias.

We also recorded the output characteristics of the WSe_2_/MoS_2_ junction for both ‘Device 1’ and ‘Device 2’ which are shown in Fig. 3a. These curves were extracted at room temperature for the condition that both gates are grounded ($V_{BG} = V_{TG} = 0$ V). A polarity convention for the drain-source bias ($V_{DS}$) was used throughout all the devices, where the drain terminal was connected to the WSe_2_ side of the junction, and the bias voltages were applied at this terminal, while the MoS_2_ side of the junction was always connected to ground. Since WSe_2_ exhibits a p-type characteristic, the junction can be treated as a forward (reverse) biased junction for positive (negative) values of $V_{DS}$. For both devices, conduction is prominent in the reverse direction while in the forward bias, the currents are relatively low. To understand this better, the relative band alignments of the individual junction regions are depicted in Fig. 3b, before the junction formation. The values for the energy gaps and electron affinities of few-layered WSe_2_ and MoS_2_ are taken from the experimental work done by Liu et al.^37^, while the graphene work function is taken from Yu et al.^38^. Figure 3c–e show the band alignment of the heterojunction post-formation under different bias ($V_{DS}$) conditions. The band diagrams for non-equilibrium conditions are strongly simplified to illustrate the relative position of the Fermi levels on either side of the heterojunction. At thermal equilibrium (Fig. 3c), the Fermi levels in WSe_2_ and MoS_2_ are aligned to the work function of graphene, which results in bending of the WSe_2_ and MoS_2_ band edges both at the graphene-semiconductor interfaces and at the WSe_2_/MoS_2_ interface. It can be seen that the SBH at the WSe_2_ side for holes is much higher than that for electrons at the MoS_2_ side. When a reverse bias is applied, depending on the magnitude of the bias, the Fermi level of WSe_2_ is at much higher energy, as seen from Fig. 3d. When the gates are off, WSe_2_ has a weak electron (minority carrier) accumulation in the conduction band (CB), and the electrons experience drift from WSe_2_-CB to MoS_2_-CB as there is no barrier at the WSe_2_/MoS_2_ interface for electrons. Additionally, electrons from the valence band (VB) of the WSe_2_ could also tunnel into the MoS_2_ CB, provided empty states exist on the MoS_2_ side. This mechanism is referred to as the band-to-band tunneling (BTBT) mechanism. If the CB of MoS_2_ is lower than the VB of WSe_2_, electrons in the VB of the WSe_2_ can tunnel through the energy window which adds an additional current component to the diffusion current in the reverse bias. For the forward bias, the MoS_2_ Fermi level is now at higher energy than that of WSe_2_, the electrons in the conduction band of MoS_2_ encounter a barrier at the WSe_2_/MoS_2_ interface and therefore, the current is limited by thermionic emission of the electrons over this barrier to pass to the WSe_2_ side of the junction. However, for ‘Device 2’ the forward bias current is much higher in comparison to ‘Device 1’ possibly due to the different lateral geometries of the graphene contacts which results in different contact resistances.

**Figure 4:**
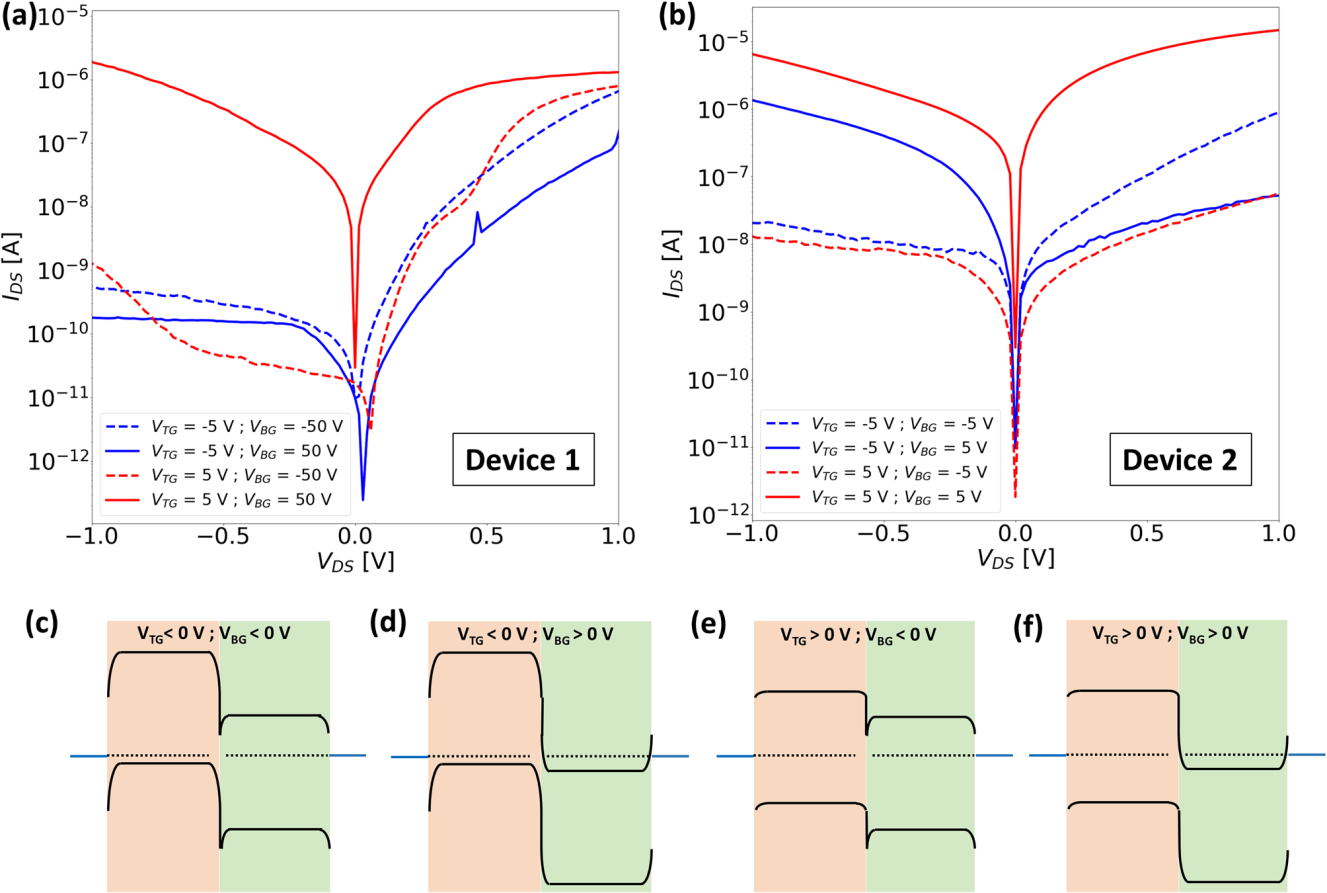
Room temperature output characteristics (semi-logarithmic) of (**a**) ‘Device 1’ and (**b**) ‘Device 2’ measured at different combinations of top and bottom gate voltages; (**c**–**f**) Corresponding band diagrams at thermal equilibrium for each type of gate configuration shown in (**a**) and (**b**). (Band diagrams for the non-equilibrium condition are illustrated in Supplementary Information-S2).

The general expression for BTBT current density from WSe_2_ to MoS_2_ can be described by the following equation^7^:

1 $$\begin{aligned} J_{BTBT} \propto \int DOS_{MoS2}(E) DOS_{WSe2}(E) \left[ f_{MoS2}(E) - f_{WSe2}(E)\right] T(E) dE \end{aligned}$$

where $DOS_{MoS2}(E)$, $DOS_{WSe2}(E)$, $f_{MoS2}(E)$ and $f_{WSe2}(E)$ correspond to the DOS and the Fermi-Dirac functions of MoS_2_ and WSe_2_ respectively. *T*(*E*) is the tunneling transmission probability which is given by the Wentzel–Kramers–Brillouin (WKB) approximation for a triangular potential barrier as follows^39^:

2 $$\begin{aligned} T_{WKB} \approx \exp \left( - \frac{4\lambda \sqrt{2 m^*} \sqrt{E_g^3}}{3q\hbar (E_g+\Delta \phi )} \right) \end{aligned}$$

where $m^*$, $E_g$, $\Delta \phi$, and $\lambda$ are the effective mass, band gap, conduction band offset, and screening length respectively. While $m^*$ and $E_g$ are determined by the material selection, the value of $\lambda$ depends on aspects such as device geometry, doping profiles, and gate capacitance^40^. Achieving a smaller $\lambda$ is crucial for effective gate modulation of the channel, necessitating the use of a high-$\kappa$ dielectric in conjunction with a thinner semiconductor body. Furthermore, it is vital to establish an abrupt doping profile at the tunneling interface, characterized by a sharp change in doping concentration across the junction. This is where 2D semiconductor heterojunctions emerge as promising alternative materials for tuning the tunneling probability.

With the help of the top and bottom gates, the carrier concentration in the semiconductor flakes could be modulated to tune the transport mechanism in these devices. This can be seen from the output characteristics of both devices measured at different combinations of polarities of the top and the bottom gate as plotted in Fig. 4a and b for ‘Device 1’ and ‘Device 2’ respectively. The band diagrams for each type of combination of top and bottom gates used for recording the output characteristics are depicted in Fig. 4c–f. It should be noted that owing to the thick EOT of ‘Device 1’ the magnitude of bottom gate bias is much higher compared to that used for ‘Device 2’. For both plots in Fig. 4 the blue (red) curves correspond to a negative (positive) polarity on the top gate while the dashed (solid) curves correspond to a negative (positive) polarity of the bottom gate. We will discuss the effect of each of the gate configurations one by one in terms of the current through the junction in both forward and reverse directions. Firstly, when both the top and bottom gates are negatively biased, both MoS_2_ and WSe_2_ should experience a strong depletion of electrons, but at the same time holes should accumulate in the WSe_2_ flake as indicated by the distance between the Fermi level and the valence band in Fig. 4c. For both devices, a significant current is observed in either direction with a relatively higher forward bias current. In this configuration, the junction resembles a typical p-n junction where the higher forward current is due to the recombination of carriers. However, in reverse bias, the possibility of elastic BTBT current should not be neglected which depends on the steepness of the band bending at the WSe_2_/MoS_2_ interface which in turn is highly dependent on the efficiency of the gates. Therefore, a slightly higher reverse bias current is observed for ‘Device 2’ where the bottom gate has a much stronger influence. Now, if the polarity of the bottom gate is reversed while keeping the top gate at a negative bias, the junction can be assumed to behave as a p+/n+ diode, where many holes accumulate in WSe_2_ and many electrons accumulate in MoS_2_ as shown in the band diagram of Fig. 4d. In the case of reverse bias, electrons tunnel from the VB of WSe_2_ to the CB of MoS_2_. One should expect a change in the direction of tunneling for a small forward bias until the Fermi level of WSe_2_ overlaps with the CB of the MoS_2_, resulting in a formation of a negative differential resistance (NDR) region, which is not observed at room temperature for our devices (discussed later in detail). For a larger forward bias, the tunneling is stopped resulting in a valley, and then with increasing forward bias, the current starts to increase owing to the majority carrier drift due to the lowering of the energy barrier for electrons at the interface with increasing forward bias. For ‘Device 1’, very low currents are observed in the reverse bias, which is again attributed to the poor modulation due to the bottom gate, which suppresses the tunneling current. But for ‘Device 2’, a much higher tunneling current is observed. On swapping the polarities of both gates, now MoS_2_ is depleted of electrons and a weak electron accumulation will result in WSe_2_ (Fig. 4e). In this scenario, the recombination currents are suppressed owing to the increase in the distance between the WSe_2_-VB and MoS_2_-CB, and much lower currents are observed for both devices. Finally, for positively biased top and bottom gates, electrons accumulate in MoS_2_ while WSe_2_ is either intrinsic or experiences a weak accumulation depending on the magnitude of the gate voltages (since WSe_2_ requires a higher gate voltage for carrier inversion than MoS_2_). In either case, the transport occurs in both directions through majority carrier drift.

**Figure 5:**
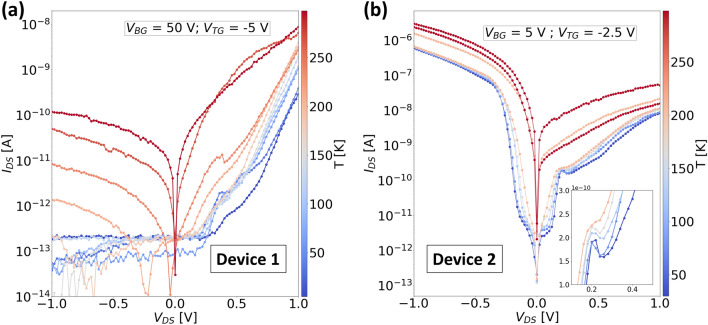
Temperature dependent output characteristics (semi-logarithmic) of (**a**) ‘Device 1’ and (**b**) ‘Device 2’ measured when $V_{TG} < 0$ V and $V_{BG} > 0$ V. The inset in sub-figure (**b**) is a magnified part of the curves in the forward bias range between 0.1 and 0.4 V.

For band-to-band tunneling in our device, the favorable bias configuration as discussed above is a positive bottom gate voltage and a negative top gate voltage. In order to characterize the transport mechanisms, we measured both ‘Device 1’ and ‘Device 2’ at cryogenic temperatures down to 20 K. The measured temperature-dependent characteristics for both devices are shown in Fig. 5. For ‘Device 1’ (Fig. 5a), a lower current is seen in the reverse bias, which is in accordance with our previous measurement at room temperature. However, this current is completely suppressed at much lower temperatures, indicating that the expected tunneling current is not dominant since the current is strongly dependent on the temperature. In the forward bias, the temperature dependence on the current is not as dominant as in the case of the reverse bias. Moreover, we observe a plateau in the current around the forward bias of 0.25–0.5 V at lower temperatures. Such behavior is attributed to the reduction of the tunneling current and masks the onset of the diffusion current thereby followed by an increase in the current. This phenomenon is a trend toward negative differential resistance (NDR), but the negative resistance is not clearly observed for this device. However, in the case of ‘Device 2’ (Fig. 5b), the NDR can be observed clearly in a similar voltage range centered around 0.2 V in the forward bias. Additionally, the current in the reverse bias is much higher indicating the presence of the expected tunneling transport mechanism, and has a much weaker temperature dependence as compared to ‘Device 1’. The NDR behavior was reproducible for different temperatures starting from 20 to 100 K. The inset in Fig. 5b shows an enlarged view of the NDR region in this temperature range, where the NDR gradually disappears at temperatures closer to room temperature because of an increase in thermionic current at higher temperatures which suppresses the tunneling current. The use of a much thinner gate dielectric in ‘Device 2’ is, therefore, a key factor that enables efficient control over the heterojunction channel in order to achieve a BTBT transport.

**Figure 6:**
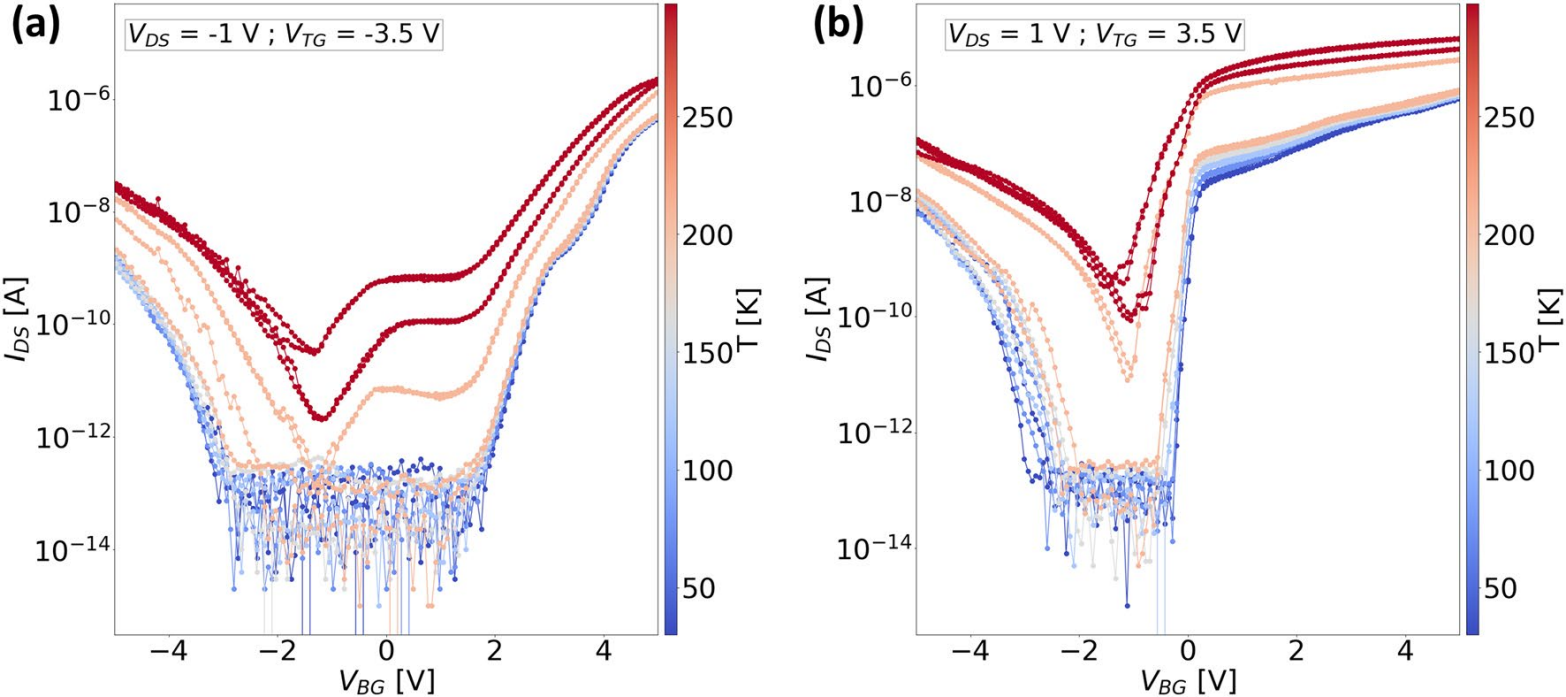
Temperature dependent transfer characteristics of ‘Device 2’ measured at (**a**) $$V_{DS} = -1 $$ V and $$V_{TG} = -3.5$$ V, (**b**) $$V_{DS} = 1 $$ V and $$V_{TG} = 3.5$$ V, by sweeping the bottom gate.

To evaluate the transistor performance of ‘Device 2’, we measured the transfer characteristics in two different bias configurations (Fig. 6a,b), where both the source-drain bias and the top gate voltage are either negative or positive. As we discussed previously, BTBT transport is normally observed for the case when the junction is in reverse bias ($$V_{DS} < 0 $$ V). The transfer curves reported so far in other works based on $$\text {WSe}_{2}$$/$$\text {MoS}_{2}$$ junctions^8–10,25^ are measured only in such a configuration, while the transfer curves for the forward bias condition are rarely shown. Here, we show the measurements of our device when the polarities are flipped, and moreover record the curves in a dual sweep to also assess the hysteresis behavior. For both bias configurations, we see ambipolar conduction with higher current levels in the n-branch, which is expected due to a stronger accumulation of electrons in $$\text {MoS}_{2}$$ with increasing bottom gate voltage. We also observed a negligible hysteresis effect in both cases owing to our h-BN dielectric environment, which slightly increases on approaching room temperature. The minimum current through the device also increases towards room temperatures ($$T > 200 $$ K), which we attribute to the increase in the thermionic current and the carrier recombination at higher temperatures. The plots of gate-leakage currents against the bottom gate voltage (Supplementary Information-S4) reveal that the leakage currents from both the top and bottom gate are quite low ($$\approx 0.1 - 10 $$ pA) and do not influence the increase in the off-currents at higher temperatures.

For negative bias configuration (Fig. 6a), the top gate and the source-drain bias remain under a negative bias while the bottom gate is swept from $-5$ V to 5 V. The current first decreases with increasing $V_{BG}$ until $V_{BG} \lesssim -1$ V, as the global bottom gate depletes the hole concentration in part of the $\text {WSe}_{2}$ flake which extends outside of the overlap region of $\text {WSe}_{2}$/$\text {MoS}_{2}$. The part of the $\text {WSe}_{2}$ flake in the overlap region remains unaffected as the electric field from the bottom gate is screened by the $\text {MoS}_{2}$ flake which is underneath the $\text {WSe}_{2}$ flake. Simultaneously, the entire $\text {MoS}_{2}$ flake experiences a strong depletion of electrons for $V_{BG} \lesssim -1$ V which increases the resistance of the device and, therefore, the currents are much lower. On increasing the $V_{BG}$ above $-1$ V, there is an increase in the current at first followed by a slight decrease which is only seen at temperatures greater than 200 K. This behavior is similar to the negative transconductance peak seen in the transfer curves obtained from the heterojunction transistor of ‘Device 1’ (Fig. 2a). With increasing $V_{BG}$, the $\text {MoS}_{2}$ flake gradually accumulates electrons which results in an initial increase in the current up to the point when $V_{BG} \approx 0$ V. Around this point, the $\text {WSe}_{2}$ is in strong depletion of holes which causes the current to decrease slightly. On further increasing the $V_{BG}$ above 2 V, both the flakes have electron accumulation which leads to a continuous increase of current with increasing $V_{BG}$. The negative transconductance behavior is suppressed at lower temperatures ($T<200$) due to the reduction of the thermionic current as discussed above.

In the positive bias configuration (Fig. 6b), a similar response as seen previously for the negative bias configuration is observed on increasing the $V_{BG}$ to about $-1$ V. However, on further increasing the $V_{BG}$, the negative transconductance is not observed and the current immediately starts to increase with increasing $V_{BG}$. We hypothesize that this is probably because the barrier for electron injection from graphene to $\text {WSe}_{2}$ is much lower in this case due to the positively biased top gate which partly overlaps the graphene /$\text {WSe}_{2}$ junction and facilitates a much better injection of electrons as compared to the case when the top gate is negatively biased. Although a much steeper switching is observed for the n-branch in the positive bias configuration, the dominant transport mechanism for this case is due to the drift-diffusion of carriers irrespective of the applied polarity of the bottom gate. In contrast, for negative bias configuration, the conduction mechanism changes from drift-diffusion to BTBT for positive bottom gate voltages. In order to confirm our hypothesis, we fabricated ‘Device 3’ with three distinct top gates as shown in the schematic in Fig. 1c, so that we can evaluate the transport in different parts of our device with respect to the electric field induced locally by the individual top gates.

**Figure 7:**
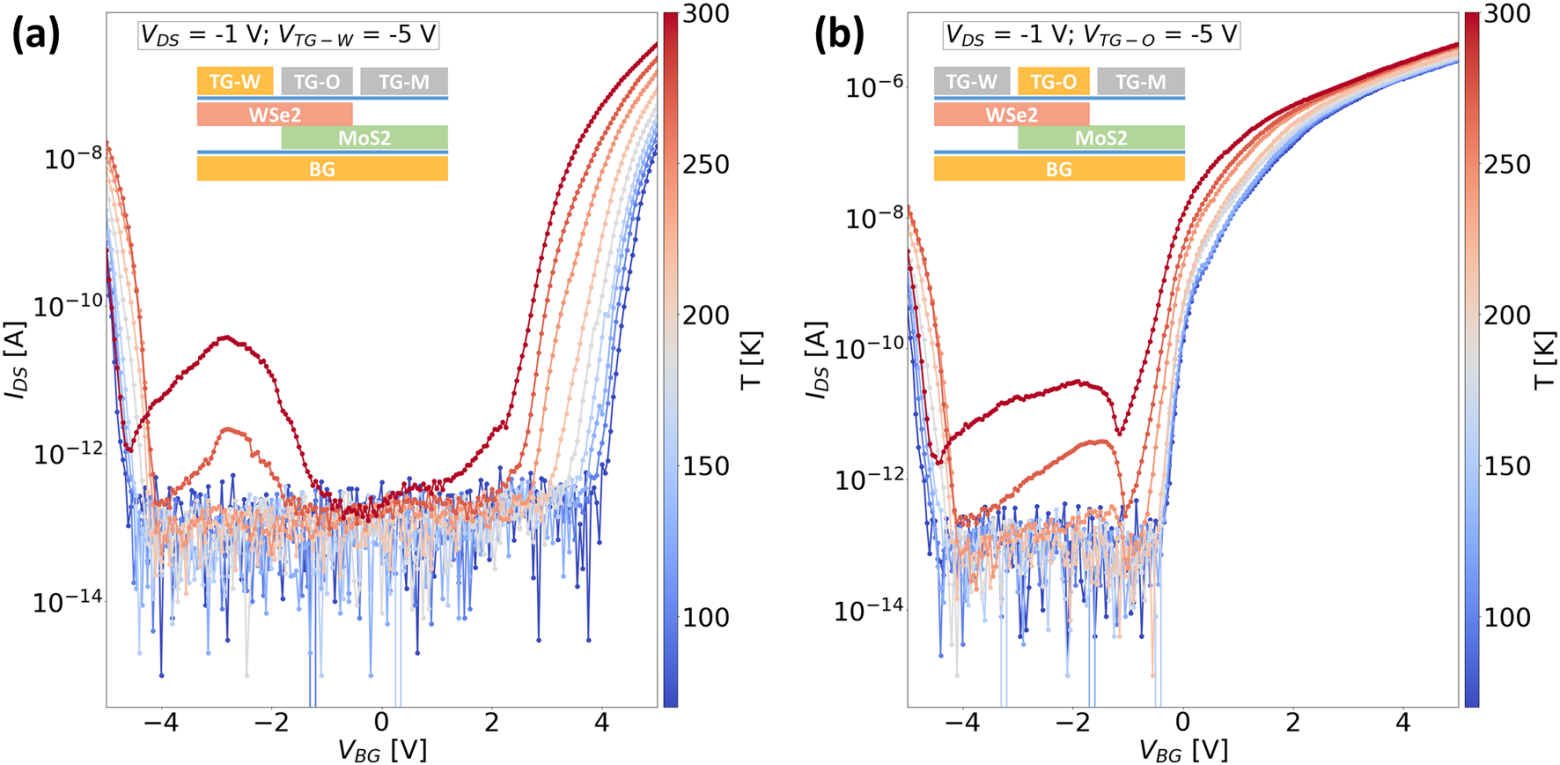
Temperature dependent transfer characteristics of ‘Device 3’ measured in negative bias configuration ($$V_{DS} = -1 $$ V and $$V_{TG} = -5$$) using (**a**) top gate covering the $$\text {WSe}_{2}$$ and $$\text {WSe}_{2}$$/graphene junction ($$V_{TG-W}$$) (**b**) top gate spanning over the $$\text {WSe}_{2}$$/$$\text {MoS}_{2}$$ overlap region ($$V_{TG-O}$$). The insets in both the figures show simplified cross-sectional device schematics indicating the bottom gate and the active top gate in yellow color and the grounded top gates in grey color.

**Figure 8:**
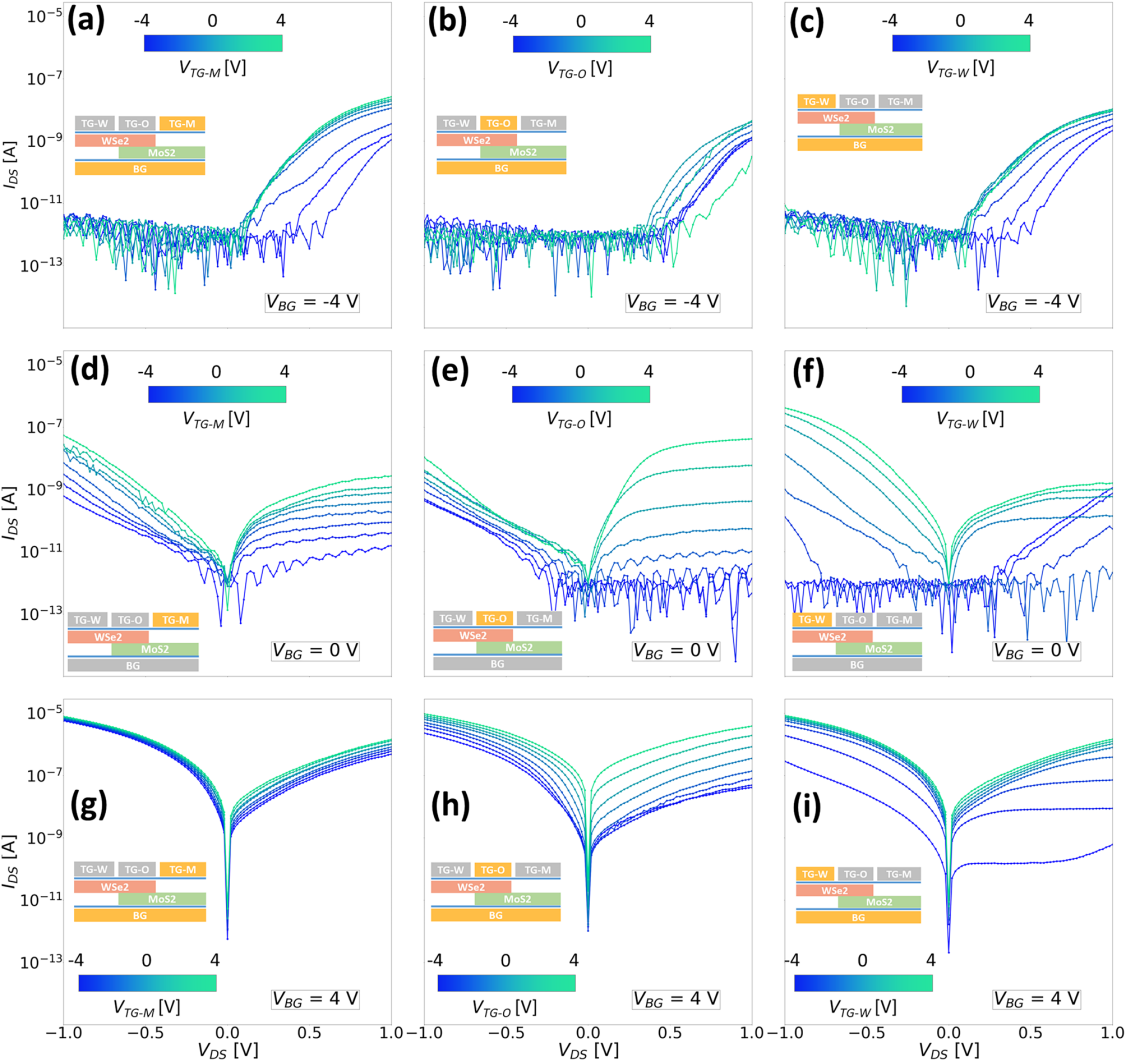
Room temperature output characteristics (semi-logarithmic) of ‘Device 3’ measured when (**a**–**c**) $$V_{BG} = -4 $$ V using $$V_{TG-M}$$, $$V_{TG-O}$$ and $$V_{TG-W}$$ respectively; (**d**–**f**) $$V_{BG} = 0 $$ V using $$V_{TG-M}$$, $$V_{TG-O}$$ and $$V_{TG-W}$$ respectively; (**g**–**i**) $$V_{BG} = 4 $$ V using $$V_{TG-M}$$, $$V_{TG-O}$$ and $$V_{TG-W}$$ respectively. The insets in both figures show a simplified cross-sectional device schematic indicating the active gates in yellow color and the grounded top gates in grey color.

We will first focus specifically on the negative bias configuration for ‘Device 3’, where we bias only one of the top gates to measure the bottom gate dependent transfer characteristics as shown in Fig. 7. We measured the device using only the top gate covering the $$\text {WSe}_{2}$$ flake which does not overlap with the $$\text {MoS}_{2}$$ flake and denote this gate by $$V_{TG-W}$$ (Fig. 7a). This top gate also overlaps the $$\text {WSe}_{2}$$/graphene junction similar to the single top gate on the entire $$\text {WSe}_{2}$$ flake as in the case of ‘Device 2’. The other two top gates are grounded as shown in the inset in grey color. The electrical response is quite similar to that of ‘Device 2’ measured in the same configuration (Fig. 6a), apart from the much wider region of carrier depletion since the $$\text {WSe}_{2}$$ in the overlap region is not modulated by the top gate. On comparing this to the case when the top gate in the overlap region ($$V_{TG-O}$$) is active, we observe much lower threshold voltages for the n-branch ($$V_{BG} > -1 $$ V) as shown in Fig. 7b. This indicates that our assumption of the lower barrier for electron transport is valid indeed, as the top gate in the overlap region does not influence the $$\text {WSe}_{2}$$/graphene barrier, unlike the case when $$V_{TG-W}$$ is active. It is worth pointing out that the presence of the NTC peak for negative bottom gate voltages should not be confused with the NDR effect. The former was observed consistently across all our devices at temperatures closer to room temperature (T > 200 K) in the transfer curves while the latter was seen in the case of output curves at low temperatures (T < 100 K). The degree of this peak is influenced by the bias condition on the top gate, where a more negative bias on the top gate leads to a higher accumulation of holes in $$\text {WSe}_{2}$$ and simultaneously $$\text {MoS}_{2}$$ experiences depletion of electrons due to a negative bottom gate bias. Therefore, with appropriate gating architectures and bias conditions, the barriers at the graphene/TMD interface as well as the tunneling across the $$\text {WSe}_{2}$$/$$\text {MoS}_{2}$$ interface can be tuned accordingly to achieve a particular type of device operation. To illustrate this, we plotted the logarithmic output characteristics of this device in all possible bias configurations in Fig. 8.

Each of the top gates, i.e $$V_{TG-M}$$, $$V_{TG-O}$$ and $$V_{TG-W}$$ are individually swept from – 4 V to 4 V while the bottom gate is biased either at – 4 V, 0 V or 4 V. When the bottom gate is negatively biased (Fig. 8a–c; $$V_{BG} = -4 $$ V), it can be seen that irrespective of which top gate is active, the device characteristics are quite similar in the sense that the device conducts only in the forward direction ($$V_{DS}>0$$ V). The negative polarity of the bottom gate depletes the electrons in $$\text {MoS}_{2}$$ and the region of $$\text {WSe}_{2}$$ extending outside the overlap region. As discussed previously for the case of ‘Device 1’ and ‘Device 2’, conduction in reverse bias is not expected for a negative polarity on the bottom gate (Fig. 4) and the results are in good agreement. This again shows that the $$\text {MoS}_{2}$$ does not experience a strong depletion even under a high negative bias, owing to a high effective n-doping (Fig. 2a).

When the bottom gate is now grounded (Fig. 8d–f; $$V_{BG} = 0 $$ V), the effect of each individual top gate can be seen distinctively. The top gate covering only the $$\text {MoS}_{2}$$ part of the device ($$V_{TG-M}$$) modulates the resistance of this region of the device as well as the graphene/$$\text {MoS}_{2}$$ barrier, where the current increases by a couple of orders of magnitude with increasing steps of $$V_{TG-M}$$ (Fig. 8d) due to higher accumulation of electrons. With the help of the overlap top gate ($$V_{TG-O}$$), the forward bias currents could be tuned more effectively, while the reverse bias currents do not show a significant change. This indicates that for negative $$V_{TG-O}$$, the transport of holes is suppressed in the forward bias due to the presence of a large barrier at the $$\text {WSe}_{2}$$/graphene interface. For positive $$V_{TG-O}$$, both electron and hole tunneling is favored in the reverse bias, but drift-diffusion is only possible for electrons in the forward bias. This behavior is almost reversed for the case when $$V_{TG-W}$$ is active owing to the effective modulation of the Schottky barrier at the $$\text {WSe}_{2}$$/graphene as seen from Fig. 8f.

Finally, when the bottom gate is at a positive bias (Fig. 8g–i; $$V_{BG} = 4 $$ V), the entire $$\text {MoS}_{2}$$ flake already has a strong electron accumulation and the effect of the additional $$V_{TG-M}$$ can be barely seen since the top gate on $$\text {MoS}_{2}$$ side does not modulate the entire $$\text {MoS}_{2}$$ flake. Therefore, the current levels obtained are also relatively higher due to a steeper bending of the band edges at the $$\text {WSe}_{2}$$/$$\text {MoS}_{2}$$ interface, which can be further tuned either by $$V_{TG-O}$$ or $$V_{TG-W}$$.

It should be noted that despite the variations in the device geometries and the slight thickness variations between ‘Device 2’ and ‘Device 3’ the electrical measurements show similar trends when measured using the same bias conditions showing the reproducible nature of the device performance. Our extensive electrical characterization of ‘Device 3’ shows that the transport properties are greatly affected by the position and the design of the top gate especially on the $\text {WSe}_{2}$ side of the junction. In order to achieve high tunneling currents, the top gate should be designed such that it covers the $\text {WSe}_{2}$ flake in the overlap region as well as the extension outside the overlap region but at the same time should exclude the region of the $\text {WSe}_{2}$/graphene interface.

Another interesting aspect in terms of the device geometry is the possibility of the formation of lateral heterojunctions. With recent developments in the one-pot growth of 2D heterojunctions^41–43^ such as $\text {WS}_{2}$/$\text {MoS}_{2}$ and $\text {WSe}_{2}$/$\text {MoSe}_{2}$, large-scale production, scalability and device-to-device variations for heterojunctions can be greatly improved. While there exists a minor constraint wherein one of the materials in the heterojunction must share either the same chalcogen or the same transition metal atom, the ability to strategically engineer band alignments through the selection of suitable materials effectively surpasses this limitation. On comparing the nature of the junction formed in lateral and quasi-lateral geometries, the heterojunction interface is covalently bonded in lateral heterojunctions, while in a quasi-lateral geometry the two semiconductors have van der Waals coupling between them. For devices based on band-to-band tunneling, this is a critical factor in determining the probability of tunneling transmission where a defect-free and high quality interface is desired, and therefore, quasi-lateral geometries are preferred. However, lateral heterojunctions are highly promising for novel electronic and opto-electronic devices based on conventional charge transport mechanisms.

The primary goal of optimizing the heterojunction devices presented in this work was initially focused on the optimization of tunnel field effect transistors (TFETs). However, due to the relatively low subthreshold swings achieved, their suitability as TFETs is limited. Nonetheless, we have recently demonstrated the successful application of the architecture shown in ’Device 2’ for a 2D diode-based temperature sensor^44^. Additionally, we have also implemented a proof-of-concept switchable bi-directional diode^45^ using ’Device 3’ for AC/DC conversion, which has the potential to benefit the design of analog circuits utilizing 2D materials.

## Conclusion

In conclusion, we have demonstrated band-to-band tunneling and negative differential resistance phenomenon in heterojunctions built entirely using 2D materials. Although the transistor performance of these devices does not show extremely steep switching characteristics, we believe that coupling ultra-thin hBN with a high-$\kappa$ dielectric material would provide the required stronger gate control to improve the switching performance. With the help of the triple gate architecture, we found that the Schottky barrier at the contact interface could be efficiently tuned in order to achieve higher tunneling currents. The electrical characteristics of this device also provide a much deeper insight into appropriate gate implementations for such heterojunction devices where the nature of the gate overlap/underlap for the heterojunction determines the effective tunneling currents. Finally, such a device architecture offers a platform to investigate relatively unstable material systems which opens up the possibility of flexible bandgap engineering.

## Methods

The 2D materials are isolated from their commercially available bulk crystals with the help of mechanical exfoliation. A scotch tape is used to peel off thin layers from the bulk crystal and these thin layers are then transferred onto PDMS substrates with a thickness of about 0.25 mm. The assembly of heterostructures is done in a nitrogen-filled glove-box environment with the help of a micromanipulator setup that essentially consists of an optical microscope, a stage, and a manipulator arm. The materials are stacked together using the dry stamping technique technique^46^ on a highly p-doped Silicon substrate with an oxide thickness of 285 nm. The source, drain, and gate contacts consisting of a 25 nm/100 nm Ni/Au metal stack are patterned using electron beam lithography (Raith150Two) with the help of a double layer resist recipe (EL 11, 950K PMMA A4). The fabricated samples are then measured in a Lakeshore CPX-VF cryostat chamber using an Agilent 4156C semiconductor parameter analyzer. The temperature-dependent measurements were carried out by first cooling the chamber down to the base temperature ($\approx$ 10 K) using liquid helium, followed by increasing temperature steps at which the measurements are performed.

### Supplementary Information

Supplementary Information.

## Data Availability

The datasets generated and analysed during the current study are available from the corresponding author on reasonable request.

## References

[1] Li, Y., Kuang, G., Jiao, Z., Yao, L. & Duan, R. Recent progress on the mechanical exfoliation of 2D transition metal dichalcogenides. *Mater. Res. Express* (2022).

[2] Novoselov K, Mishchenko OA, Carvalho OA, Castro Neto A (2016). 2D materials and van der Waals heterostructures. Science.

[3] Lemme MC, Akinwande D, Huyghebaert C, Stampfer C (2022). 2D materials for future heterogeneous electronics. Nat. Commun..

[4] Hassan, J. Z. *et al.* 2D material-based sensing devices: An update. *J. Mater. Chem. A* (2023).

[5] Fei W, Trommer J, Lemme MC, Mikolajick T, Heinzig A (2022). Emerging reconfigurable electronic devices based on two-dimensional materials: A review. InfoMat.

[6] Chava P, Fekri Z, Vekariya Y, Mikolajick T, Erbe A (2023). Band-to-band tunneling switches based on two-dimensional van der Waals heterojunctions. Appl. Phys. Rev..

[7] Guo Z (2018). Independent band modulation in 2D van der Waals heterostructures via a novel device architecture. Adv. Sci..

[8] He J (2018). 2D tunnel field effect transistors (FETs) with a stable charge-transfer-type p+ WSe_2_ source. Adv. Electron. Mater..

[9] Jeon HB, Shin GH, Lee KJ, Choi S-Y (2020). Vertical-tunneling field-effect transistor based on WSe_2_–MoS_2_ heterostructure with ion gel dielectric. Adv. Electron. Mater..

[10] Nakamura K (2020). All 2D heterostructure tunnel field-effect transistors: Impact of band alignment and heterointerface quality. ACS Appl. Mater. Interfaces.

[11] Na J, Kim Y, Smet JH, Burghard M, Kern K (2019). Gate-tunable tunneling transistor based on a thin black phosphorus-SnSe_2_ heterostructure. ACS Appl. Mater. Interfaces.

[12] Oliva N (2020). WSe_2_/SnSe_2_ vdw heterojunction tunnel FET with subthermionic characteristic and MOSFET co-integrated on same WSe_2_ flake. NPJ 2D Mater. Appl..

[13] Yang S-H (2019). Atomically thin van der Waals tunnel field-effect transistors and its potential for applications. Nanotechnology.

[14] Yan X (2017). Tunable SnSe_2_/WSe_2_ heterostructure tunneling field effect transistor. Small.

[15] Xu J, Jia J, Lai S, Ju J, Lee S (2017). Tunneling field effect transistor integrated with black phosphorus-MoS_2_ junction and ion gel dielectric. Appl. Phys. Lett..

[16] Wang J (2018). Vertical WS_2_/SnS_2_ van der Waals heterostructure for tunneling transistors. Sci. Rep..

[17] Sato Y (2021). Intrinsic electronic transport properties and carrier densities in PtS_2_ and SnSe_2_: Exploration of n+-source for 2D tunnel FETs. Adv. Electron. Mater..

[18] Lv Q (2020). Interlayer band-to-band tunneling and negative differential resistance in van der Waals BP/InSe field-effect transistors. Adv. Funct. Mater..

[19] Liu X (2017). Modulation of quantum tunneling via a vertical two-dimensional black phosphorus and molybdenum disulfide p–n junction. ACS Nano.

[20] Lim SK (2018). Operation mechanism of a MoS_2_/BP heterojunction FET. Nanomaterials.

[21] Koo B, Shin GH, Park H, Kim H, Choi S-Y (2018). Vertical-tunneling field-effect transistor based on MoTe_2_/MoS_2_ 2d–2d heterojunction. J. Phys. D.

[22] Li W, Xiao X, Xu H (2019). Versatile electronic devices based on WSe_2_/SnSe_2_ vertical van der Waals heterostructures. ACS Appl. Mater. Interfaces.

[23] Wang X, Sun Y, Liu K (2019). Chemical and structural stability of 2D layered materials. 2D Materials.

[24] Roy T (2015). Dual-gated MoS_2_/WSe_2_ van der Waals tunnel diodes and transistors. ACS Nano.

[25] Nourbakhsh A, Zubair A, Dresselhaus MS, Palacios T (2016). Transport properties of a MoS_2_/WSe_2_ heterojunction transistor and its potential for application. Nano Lett..

[26] Roy T (2014). Field-effect transistors built from all two-dimensional material components. ACS Nano.

[27] Yang M (2022). Charge transport behaviors in a multi-gated WSe_2_/MoS_2_ heterojunction. Appl. Phys. Lett..

[28] Chava, P. *et al.* Tunneling transport in WSe_2_–MoS_2_ heterojunction transistor enabled by a two-dimensional device architecture. in *2022 Device Research Conference (DRC)*, 1–2 (IEEE, 2022).

[29] Splendiani A (2010). Emerging photoluminescence in monolayer MoS_2_. Nano Lett..

[30] Zheng Y, Gao J, Han C, Chen W (2021). Ohmic contact engineering for two-dimensional materials. Cell Rep. Phys. Sci..

[31] Dean CR (2010). Boron nitride substrates for high-quality graphene electronics. Nat. Nanotechnol..

[32] Li C (2017). WSe_2_/MoS_2_ and MoTe_2_/SnSe_2_ van der Waals heterostructure transistors with different band alignment. Nanotechnology.

[33] Sun X (2021). Visualizing band profiles of gate-tunable junctions in MoS_2_/WSe_2_ heterostructure transistors. ACS Nano.

[34] Wu D (2019). Visualization of local conductance in MoS_2_/WSe_2_ heterostructure transistors. Nano Lett..

[35] Patoary NH (2023). Improvements in 2d p-type WSe_2_ transistors towards ultimate CMOS scaling. Sci. Rep..

[36] Lee I (2016). Gate-tunable hole and electron carrier transport in atomically thin dual-channel WSe_2_/MoS_2_ heterostructure for ambipolar field-effect transistors. Adv. Mater..

[37] Liu L (2022). Tunable current regulative diode based on van der Waals stacked MoS_2_/WSe_2_ heterojunction-channel field-effect transistor. Adv. Electron. Mater..

[38] Yu Y-J (2009). Tuning the graphene work function by electric field effect. Nano Lett..

[39] Griffiths DJ, Schroeter DF (2018). Introduction to Quantum Mechanics.

[40] Seabaugh AC, Zhang Q (2010). Low-voltage tunnel transistors for beyond CMOS logic. Proc. IEEE.

[41] Sahoo PK, Memaran S, Xin Y, Balicas L, Gutiérrez HR (2018). One-pot growth of two-dimensional lateral heterostructures via sequential edge-epitaxy. Nature.

[42] Sahoo PK (2019). Bilayer lateral heterostructures of transition-metal dichalcogenides and their optoelectronic response. ACS Nano.

[43] Berweger S (2020). Spatially resolved persistent photoconductivity in MoS_2_–WS_2_ lateral heterostructures. ACS Nano.

[44] Matthus, C. D. *et al.* Ivt characteristics and temperature sensor performance of a fully-2d WSe_2_/MoS_2_ heterojunction diode at cryogenic temperatures. *IEEE J. Electron. Dev. Soc.* (2023).

[45] Matthus, C. D. *et al.* 2d bdiode: A switchable bidirectional diode for analog electronic circuits fabricated entirely from 2d materials. in *Micro and Nanoengineering Conference 2023* (2023).

[46] Castellanos-Gomez A (2014). Deterministic transfer of two-dimensional materials by all-dry viscoelastic stamping. 2D Mater..

