# Supplementary material for: Electrical characterization of multi-gated WSe2/MoS2 van der Waals heterojunctions
Source: Sci Rep. 2024 Mar 9;14:5813. doi: 10.1038/s41598-024-56455-x (PMC10925069; doi:10.1038/s41598-024-56455-x)
Supplement: Supplementary file 1 — Supplementary Information. [file 41598_2024_56455_MOESM1_ESM.pdf]

Supplementary Information: Electrical characterization of multi-gated WSe_2/MoS_2 van der Waals heterojunctions

Phanish Chava[1,2,*], Vaishnavi Kateel[1], Kenji Watanabe[3], Takashi Taniguchi[4], Manfred Helm[1], Thomas Mikolajick[2,5], and Artur Erbe[1,2]

[1]Institute of Ion Beam Physics and Materials Research, Helmholtz Zentrum Dresden-Rossendorf, 01328 Dresden, Germany
[2]Faculty of Electrical and Computer Engineering, Technische Universität Dresden, 01062 Dresden, Germany
[3]Research Center for Electronic and Optical Materials, National Institute for Materials Science, 1-1 Namiki, Tsukuba 305-0044, Japan
[4]Research Center for Materials Nanoarchitectonics, National Institute for Materials Science, 1-1 Namiki, Tsukuba 305-0044, Japan
[5]NaMLab gGmbH, 01187 Dresden, Germany
[*]p.chava@hzdr.de

ABSTRACT

Vertical stacking of different two-dimensional (2D) materials into van der Waals heterostructures exploits the properties of individual materials as well as their interlayer coupling, thereby exhibiting unique electrical and optical properties. Here, we study and investigate a system comprising entirely of different 2D materials for the implementation of electronic devices that are based on quantum mechanical band-to-band tunneling transport such as tunnel diodes and tunnel field-effect transistors. We fabricated and characterized van der Waals heterojunctions based on semiconducting layers of WSe_2 and MoS_2 by employing different gate configurations to analyze the transport properties of the junction. We found that the device dielectric environment is crucial for achieving tunneling transport across the heterojunction by replacing thick oxide dielectric with thin layers of hexagonal boronnitride. With the help of additional top gates implemented in different regions of the heterojunction, it was seen that the tunneling properties as well as the Schottky barriers at the contact interfaces could be tuned efficiently by using layers of graphene as an intermediate contact material.

S1. Extraction of Schottky barrier heights for MoS2 and WSe2 transistors

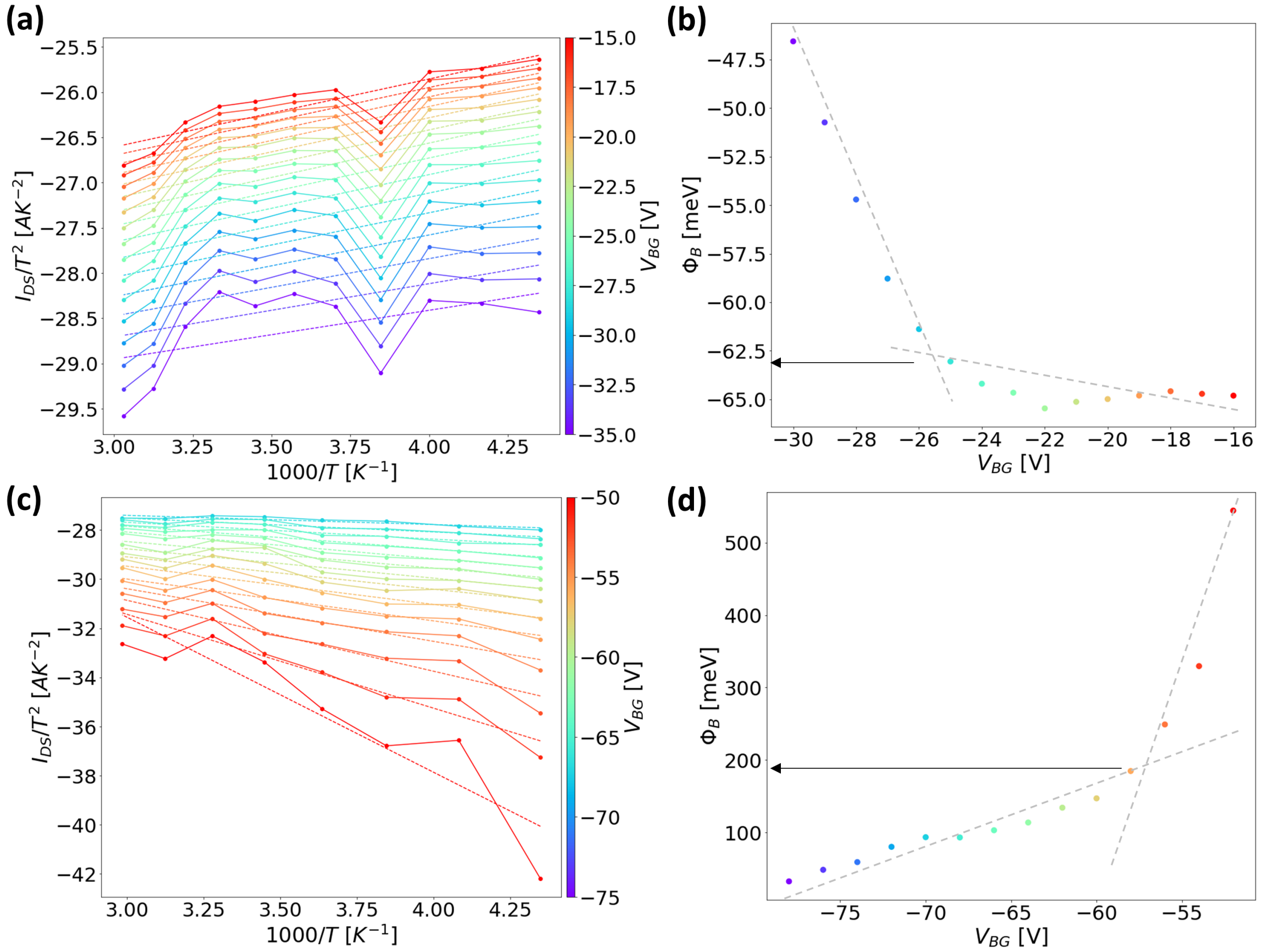

Figure S1. Schottky barrier height extraction: (a) Arrhenius plot obtained from MoS_2 transfer characteristics; (b) the slopes of the Arrhenius plot in (a) plotted against the gate voltage; (c) Arrhenius plot obtained from WSe_2 transfer characteristics; (d) the slopes of the Arrhenius plot in (c) plotted against the gate voltage.

For the Schottky barrier height (SBH) extraction, variable temperature transfer characteristics are performed in the range of 230 K - 330 K to avoid weak current in the low temperature regime (below 150 K). The Schottky barrier affects the source-drain current (I_{DS}) at the contact interface for electron or hole injection. The I_{DS} consists of thermionic emission current (I_{TIE}) crossing over the SB and tunneling current (I_{TUN}) through the Schottky barrier. The gate bias (V_{GS}) determines which of the two currents contribute to I_{DS}[1]. The voltage required to have no bending of the semiconductor band at the interface is the flat band voltage (V_{FB}) and it plays an important role in accurate SBH calculation. The current is dominated by I_{TIE} until the flat band condition is reached after which the barrier is thinned and I_{TUN} flows through the barrier[1]. The drain current flowing across a 2D channel in a transistor geometry can be given by thermionic emission theory[2] as follows:

$$I_{DS} = AA^*T^2 \exp\left(-\frac{q\phi_B}{k_BT}\right) \exp\left(\frac{qV_{DS}}{k_BT} - 1\right) \quad (1)$$

where A is the contact area, A^* is the Richardson constant, T is the absolute temperature, q is the electronic charge, V_{DS} is the source-drain bias, k_B is the Boltzmann constant and ϕ_B is the effective Schottky barrier. When $V_{DS} >> (k_BT/q)$, then an Arrhenius plot of $\ln(I_{DS}/T^2)$ vs $1/T$ is drawn as shown in Figure S1(a) and (c) to extract the Schottky barrier height (ϕ_{SBH}) using the equation[3]:

$$ln(\frac{I_{DS}}{T^2}) = ln(AA^*) - \frac{-q(\phi_B - V_D)}{k_BT} \quad (2)$$

The linear fit of the Arrhenius plot at constant drain bias has a slope of $-\frac{-q(\phi_B - V_D)}{k_B}$ and an intercept of $\ln(AA^*)$. From the slope, ϕ_B for various gate voltages is calculated and is plotted against V_{GS} as shown in Figure S1(b) and (d). The effective Schottky barrier has a linear relationship with V_{GS} until the flat band condition is reached, after which the linear dependence is lost[3]. This transition point indicates the flat band voltage as well as the Schottky barrier height.

The Schottky barrier height obtained for electrons in case of MoS_2 was extracted to be approximately 60 meV and the barrier height for holes in case of WSe_2 is approximately 200 meV, which is roughly three times higher than that of MoS_2.

S2. Additional band diagrams - Device 2

Figure S2 and Figure S3 show the non-equilibrium energy band diagrams of Device 2 under reverse ($V_{DS} < 0$ V) and forward bias ($V_{DS} > 0$ V) respectively, each indicating different configurations of top and bottom gate voltages applied to the device. It should be noted that the position of the Fermi levels in these sketches is roughly assumed throughout all the sub-figures. The effect of the global bottom gate on the WSe_2 is also slightly incorporated into the sketches, due to which the position of the Fermi level in WSe_2 also changes. For example, this can be observed from Figure S2(a)-(c), where the top gate always remains under a negative bias, but the variation of the bottom gate voltage influences the position of the Fermi level in WSe_2.

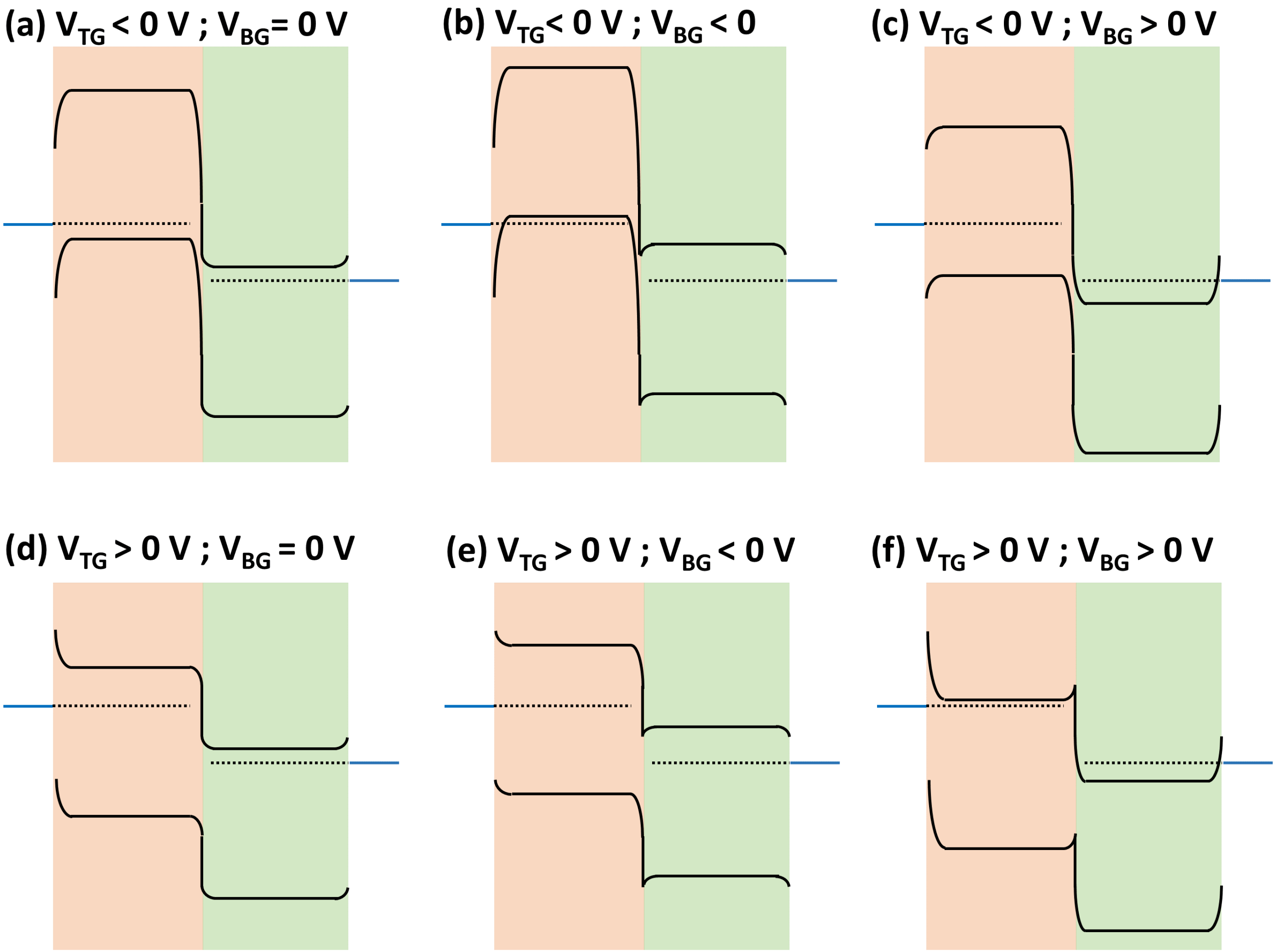

Figure S2. Energy band diagrams for Device 2 in reverse bias ($V_{DS} < 0$ V) under different top and bottom gate configurations indicating the variations in the position of Fermi level with respect to the conduction and the valence bands of WSe_2 (shown in red) and MoS_2(shown in green).

In both the cases, we can see the influence of the gates on the Schottky barrier, which is quite large for holes especially at the WSe_2 side of the junction for some bias configurations as seen in sub-figures (a) and (b) of both Figure S2 and Figure S3.

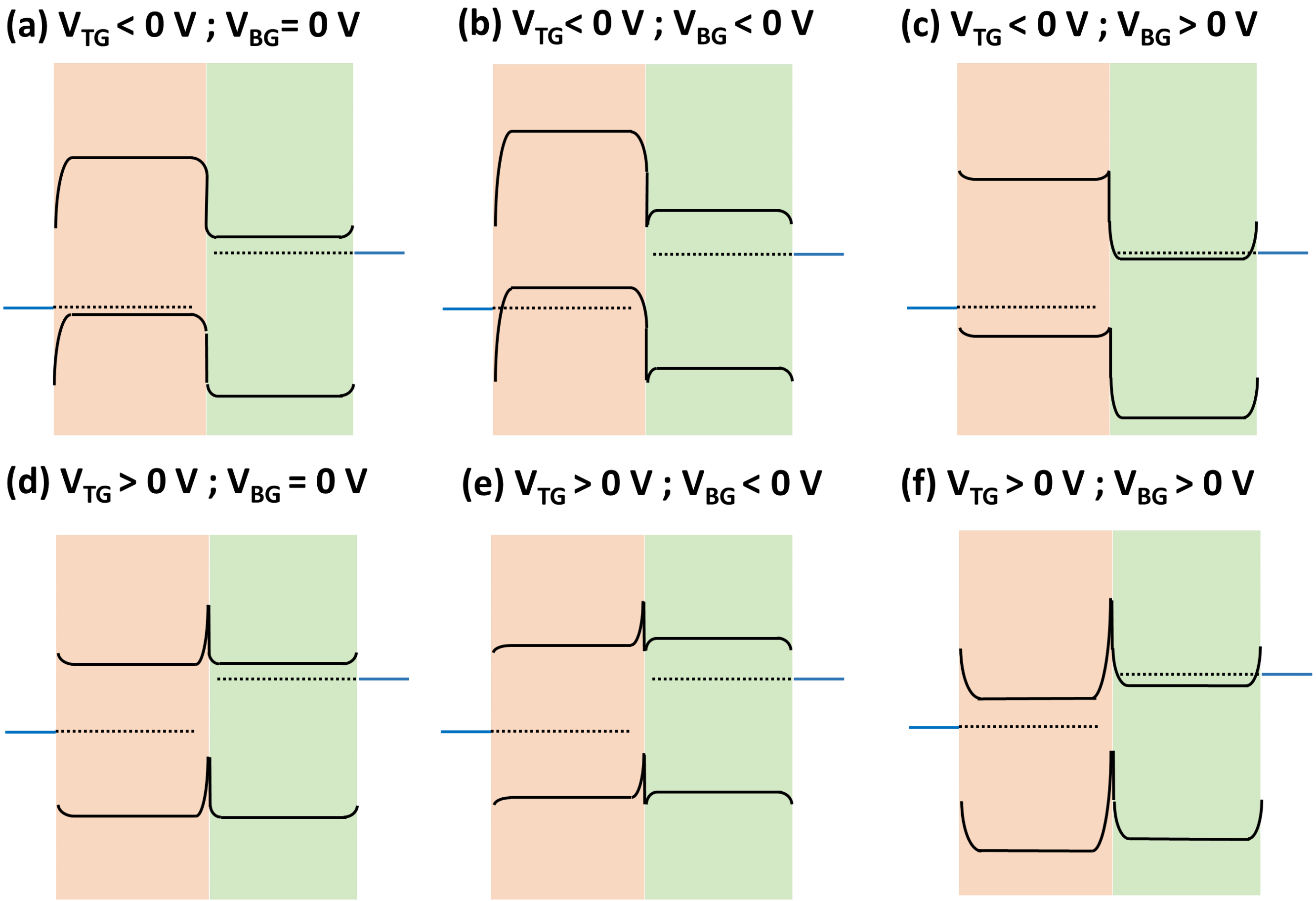

Figure S3. Energy band diagrams for Device 2 in forward bias ($V_{DS} > 0$ V) under different top and bottom gate configurations indicating the variations in the position of Fermi level with respect to the conduction and the valence bands of WSe_2 (shown in red) and MoS_2(shown in green).

S3. Extracted Subthreshold Swings - Device 2

The average Subthreshold swing values are extracted for Device 2, using a reliable extraction method that we reported previously[4]. We extracted the values for both the bias configurations for the device as seen in Figure S4, wherein each configuration has two distinct current branches. Here, we only show the extracted values for curves measured until a temperature of 150 K, since at higher temperatures the values are much higher. The obtained values for the tunneling branch, i.e the n-branch in Figure S4(a) are roughly between 300 and 400 mV/dec and show a very slight variation with temperature, while for the p-branch, the values tend to increase with temperature from 400 to 700 mV/dec. For the positive bias configuration, the swings are much lower as compared to the negative bias configuration, especially for the n-branch where the values are between 100 and 200 mV/dec, which is also predictable from the steeper transfer curves obtained in this configuration.

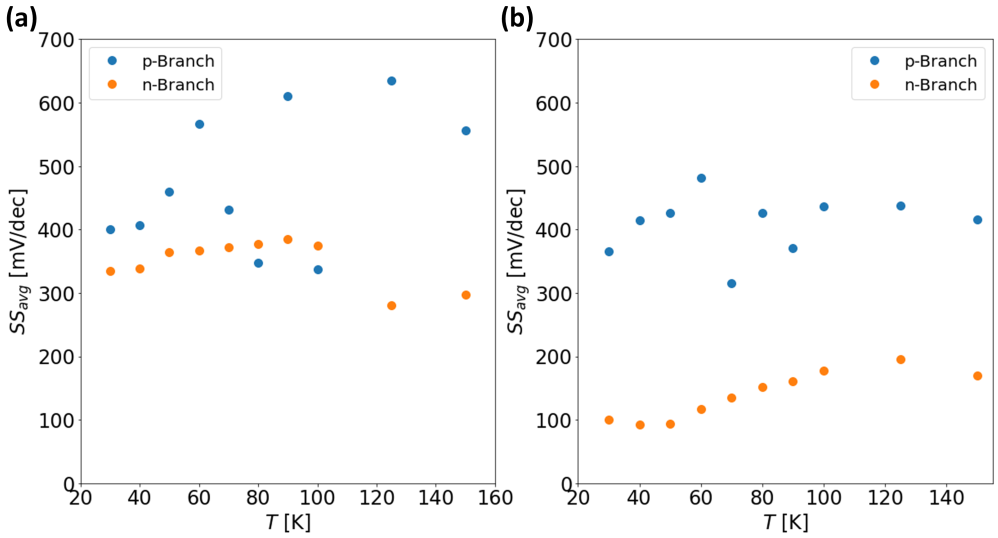

Figure S4. Average subthreshold swing (SS_{avg}) values extracted from the temperature dependent transfer curves of Device 2 for both n- and p-branches measured at (a) $V_{DS} = -1$ V and $V_{TG} = -3.5$ V (b) $V_{DS} = 1$ V and $V_{TG} = 3.5$ V.

S4. Top and Bottom Gate leakages - Device 2

The top and the bottom gate leakage currents are plotted against the bottom gate voltage while measuring the transfer curves for Device 2 shown in Figure 6 of the manuscript. In both the configurations, it can be seen that leakage from both the gates is quite low with maximum values reaching about 10 pA for the top gate and around 1 pA for the bottom gate particularly towards higher temperatures.

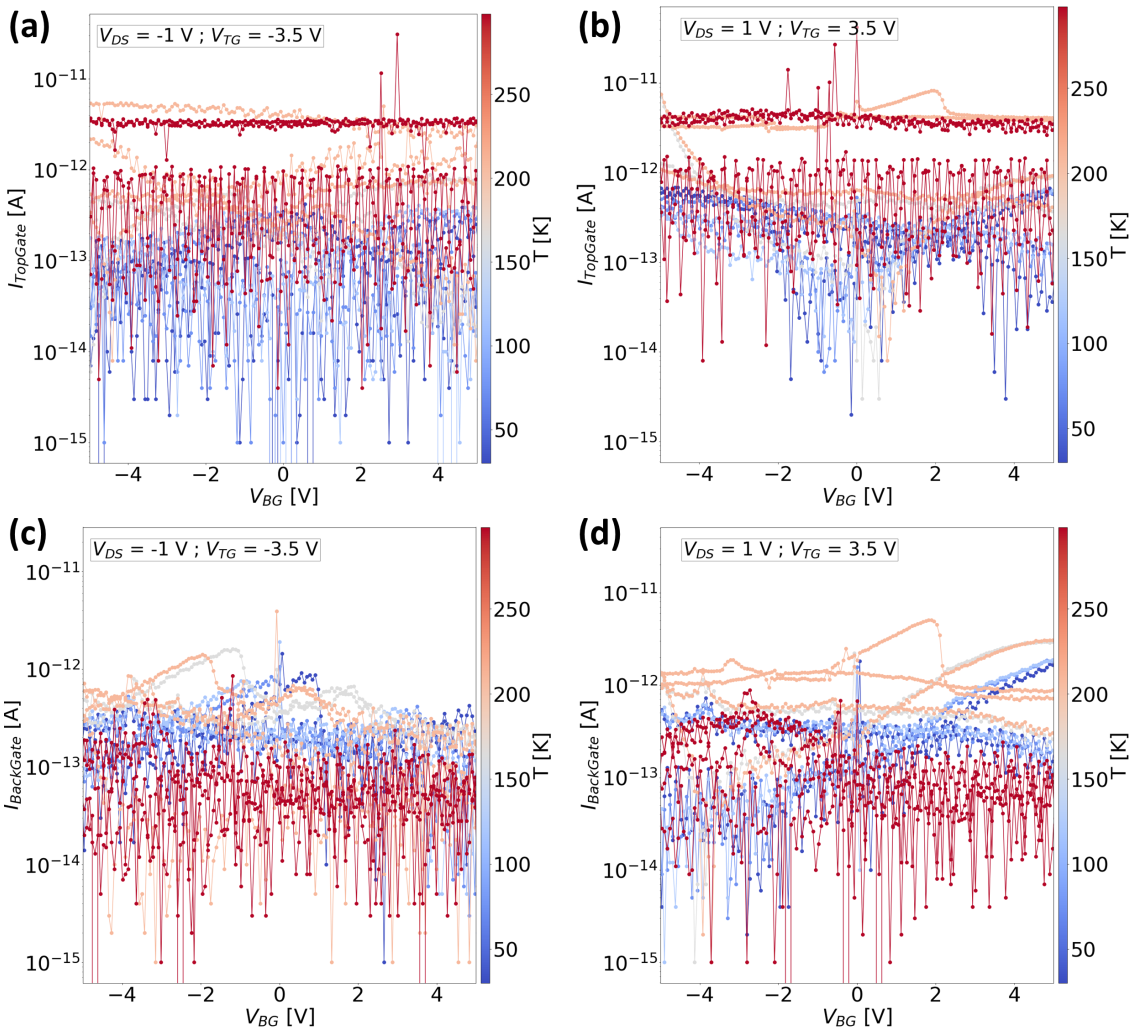

Figure S5. (a)-(b) Top and (c)-(d) bottom gate leakages recorded while measuring the temperature-dependent transfer curves of Device 2.

S5. Performance comparison of fabricated devices

Table 1 summarizes a few parameters of all the fabricated devices mentioned in the manuscript to provide a comparative overview of device performance. The distance between the graphene contact electrodes is used to extract the device's length. The width of a device is extracted as the minimum width of the TMD in the junction region. The equivalent oxide thickness (EOT) of hBN is extracted as $EOT = t_{hBN} * \varepsilon_{hBN}/3.9$, where t_{hBN} and ε_{hBN} correspond to the thickness and dielectric constant of hBN ($\varepsilon_{hBN} \approx 3.5$).

Device parameters	Device 1	Device 2	Device 3
Width [µm] x Length [µm]	0.5 x 2	3 x 10	10 x 12
WSe_2 / MoS_2 thickness [nm]	7 / 10	5 / 3	3 / 4
Top / bottom equivalent oxide thickness [nm]	12 / 298	9 / 11	6 / 8
Peak transconductance [$\mu S\, \mu m^{-1}$]	$1.4 \times 10^{-}3$	$8.5 \times 10^{-}3$	$15 \times 10^{-}3$
Transconductance efficiency [V^{-1}]	1.1	7.5	46.3
Average Subthreshold Swing - 300 K [mV/dec]	1250	400	255
Average Subthreshold Swing - 30 K [mV/dec]	-	110	87
Presence of NDR	No	Not clear	Yes
Number of fabricated devices	8	6	3
Number of functional devices	5	3	1
Device yield [%]	62.5	50	33.3

Table 1. Overview of various device parameters for the fabricated devices mentioned in the manuscript.

S6. Measurement reproducibility and device-to-device variations

Figure S6 shows the plot of transfer characteristics at room temperature for 5 heterojunction devices fabricated using the 'Device 1' architecture. The label 'Device 1' in the plot corresponds to the device mentioned in the manuscript. It can be seen that although the curves exhibit slight device-to-device variations, the general trends across all the devices are consistent. Firstly, all the devices exhibit a clear ambipolar behavior with a higher p-branch. Secondly, the presence of the negative transconductance (NTC) peak can also be observed for negative bottom gate voltages. The variations are attributed to the different device geometries owing to the irregular shapes of the mechanically exfoliated 2D flakes as well as the variation in the thicknesses of the TMDs which are in the range of 3 nm to 10 nm.

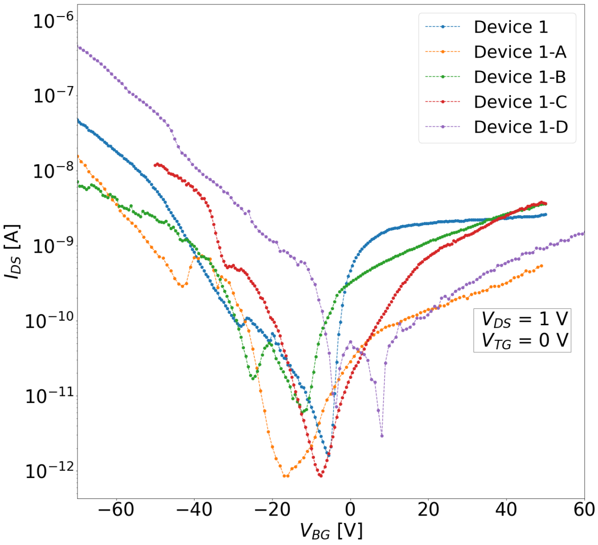

Figure S6. Room temperature transfer characteristics (semi-logarithmic) of 5 different devices fabricated using the 'Device 1' architecture.

The device-to-device variations and measurement reproducibility for devices with 'Device 2' architecture is illustrated in Figure S7 with the help of temperature-dependent output characteristics. The curves in Figure S7(a) correspond to the 'Device 2' mentioned in the manuscript. For all the subfigures, the top and the bottom voltage are set at a common bias of -2.5 V and 5 V respectively. We were able to observe reproducible measurements among these devices in terms of the room temperature transport behavior, but towards low temperature, only one device (Figure S7(a)) shows a clear negative differential resistance (NDR) effect. Unfortunately, the NDR effect was not very clearly observed for the other devices.

In Figure S8, we show the reproducible NDR effect in 'Device 2' by varying the top gate voltage from - 1.5 V to -3.5 V. It can be seen that the NDR effect is clearly seen for different biasing conditions of the top gate. This shows that the observed NDR effect is indeed valid and not merely a measurement glitch.

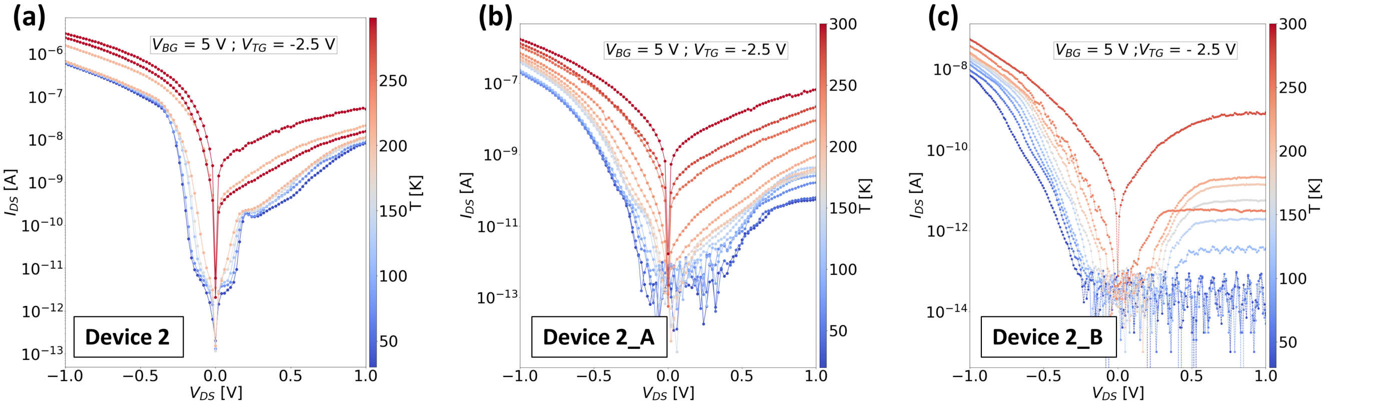

Figure S7. Temperature-dependent output characteristics (semi-logarithmic) for 3 different devices fabricated using the 'Device 2' architecture. (a) Device 2 (b) Device 2_A (c) Device 2_B

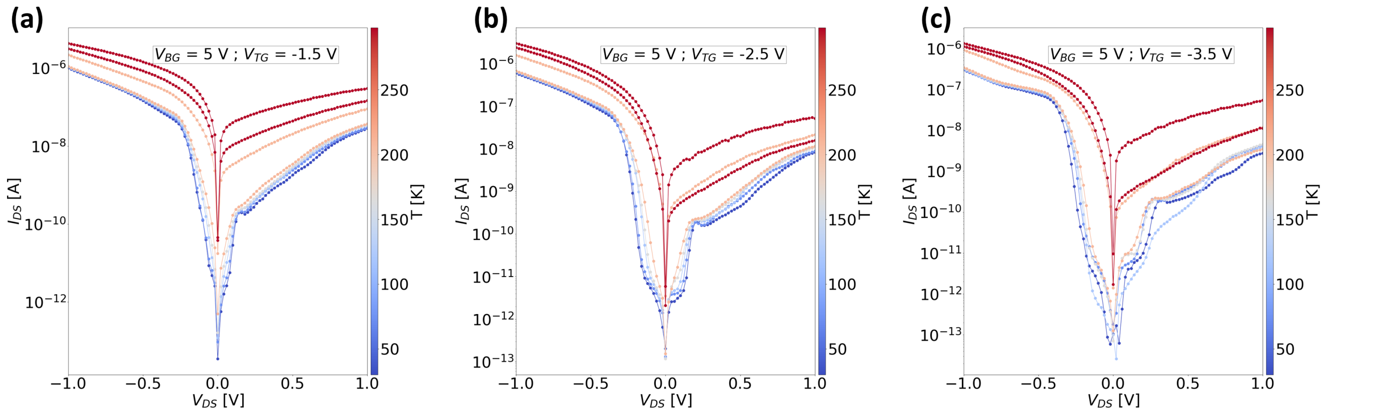

Figure S8. Temperature-dependent output characteristics (semi-logarithmic) for 'Device 2' with $V_{BG} = 5V$ and decreasing top gate voltages (a) $V_{TG} = -1.5V$, (b) $V_{TG} = -2.5V$, and (c) $V_{TG} = -3.5V$

References

1. Schulman, D. S., Arnold, A. J. & Das, S. Contact engineering for 2d materials and devices. *Chem. Soc. Rev.* **47**, 3037–3058 (2018).
2. Li, S.-L., Tsukagoshi, K., Orgiu, E. & Samorì, P. Charge transport and mobility engineering in two-dimensional transition metal chalcogenide semiconductors. *Chem. Soc. Rev.* **45**, 118–151 (2016).
3. Houssa, M., Dimoulas, A. & Molle, A. *2D materials for nanoelectronics*, vol. 17 (CRC Press, 2016).
4. Chava, P., Fekri, Z., Vekariya, Y., Mikolajick, T. & Erbe, A. Band-to-band tunneling switches based on two-dimensional van der Waals heterojunctions. *Appl. Phys. Rev.* **10**, 011318 (2023).
